# Quantitative Analysis of α-Synuclein Solubility in Living Cells Using Split GFP Complementation

**DOI:** 10.1371/journal.pone.0043505

**Published:** 2012-08-22

**Authors:** Ahmed Kothawala, Kiri Kilpatrick, Jose Andres Novoa, Laura Segatori

**Affiliations:** 1 Department of Chemical and Biomolecular Engineering, Rice University, Houston, Texas, United States of America; 2 Department of Biochemistry and Cell Biology, Rice University, Houston, Texas, United States of America; 3 Department of Bioengineering, Rice University, Houston, Texas, United States of America; Universitat Autònoma de Barcelona, Spain

## Abstract

Presently incurable, Parkinson's disease (PD) is the most common neurodegenerative movement disorder and affects 1% of the population over 60 years of age. The hallmarks of PD pathogenesis are the loss of dopaminergic neurons in the *substantia nigra pars compacta*, and the occurrence of proteinaceous cytoplasmic inclusions (Lewy bodies) in surviving neurons. Lewy bodies are mainly composed of the pre-synaptic protein alpha-synuclein (αsyn), an intrinsically unstructured, misfolding-prone protein with high propensity to aggregate. Quantifying the pool of soluble αsyn and monitoring αsyn aggregation in living cells is fundamental to study the molecular mechanisms of αsyn-induced cytotoxicity and develop therapeutic strategies to prevent αsyn aggregation. In this study, we report the use of a split GFP complementation assay to quantify αsyn solubility. Particularly, we investigated a series of naturally occurring and rationally designed αsyn variants and showed that this method can be used to study how αsyn sequence specificity affects its solubility. Furthermore, we demonstrated the utility of this assay to explore the influence of the cellular folding network on αsyn solubility. The results presented underscore the utility of the split GFP assay to quantify αsyn solubility in living cells.

## Introduction

Parkinson’s disease (PD) is the most prevalent neurodegenerative movement disorder, affecting 1% of the world’s population over the age of 60 years [1]. The hallmarks of PD pathogenesis are the loss of dopaminergic neurons in the *substantia nigra pars compacta* and the occurrence of cytoplasmic inclusions called Lewy bodies (LB) in surviving dopaminergic neurons [2]. *Post mortem* analyses revealed that the main component of LB is the pre-synaptic protein alpha-synuclein (αsyn) and of trace amounts of ubiquitin and molecular chaperones [3], suggesting that they result from the aberrant accumulation and aggregation of misfolded, undegraded αsyn. Duplications or triplications of the αsyn locus [4], [5], as well as mutations in αsyn-encoding gene - A53T, A30P & E46K – lead to increased aggregation and have been linked to familial cases of PD [6]–[10]. Overexpression of αsyn results in the formation of inclusion bodies, cytotoxicity and cell death in animal models and cell cultures [11]–[13]. Misfolding and aggregation of αsyn has been associated with impairment of proteasomal degradation, another common trait of PD pathogenesis [14]–[16]. In summary, aberrant accumulation of misfolded αsyn plays a key role in development of PD pathogenesis. Therefore, monitoring αsyn aggregation in living cells in a quantitative fashion is important to study the molecular mechanisms associated with αsyn-induced cytotoxicity and develop therapeutic strategies for the treatment of PD.

A number of αsyn variants containing mutations that alter the protein’s rate of aggregation have been characterized [6]–[9]. Among mutations linked to familial cases of PD, the A53T αsyn variant was shown to aggregate at a much faster rate than wt αsyn in cell cultures and *in vitro* [9], [10], [17]. C-terminal truncations have also been reported to aggregate at higher rates than wt αsyn [18]–[20], demonstrating that the proline-rich C-terminal region plays a fundamental role in limiting αsyn misfolding and aggregation [18]–[22]. A recent study demonstrated that a truncation variant of αsyn consisting of amino acids 1–123 (αsyn123) readily formed aggregates *in vitro* [19]. Interestingly, it was also shown that truncated αsyn accumulates in LB [23], suggesting that lower molecular weight truncated αsyn species may have a role in PD pathology. Recently, in an effort to decipher the determinants of αsyn aggregation, a rationally designed mutant containing three proline substitutions (TP αsyn, containing substitutions A30P, A56P and A76P) was also constructed and demonstrated to resist aggregation *in vitro* [24]. Its solubility in cell cultures, however, is not known.

A number of methods to study αsyn aggregation *in vitro* have been reported and include microscopy [21], size-exclusion chromatography [25], and NMR spectroscopy [26]. These techniques rely on the use of purified proteins for analysis. Hence, they preclude the study of αsyn aggregation in living cells, which is necessary to decipher the pathogenic mechanisms that lead to increased levels of misfolded and aggregated αsyn and to identify gene targets for therapy.

Microscopy based techniques have been used to monitor protein aggregation in living cells [27], [28]. Particularly, αsyn aggregation can be detected using αsyn-specific antibodies [11], [29] or by overexpressing αsyn variants fused to fluorescent reporters such as GFP [17], [30], [31]. The main limitation of using GFP fusions as aggregation reporters is that aggregation events that occur after the formation of the GFP chromophore do not alter fluorescence emission, leading to detection of GFP fluorescence irrespective of αsyn aggregation state. To overcome this limitation, techniques that rely on fluorescence complementation have been developed. Particularly, αsyn was fused to non-fluorescent complementary GFP fragments and the resulting fusion molecules were co-expressed in mammalian cells. αsyn self-association causes close proximity of the two GFP fragments and results in bimolecular fluorescence complementation (BiFC). Hence, the intensity of the fluorescence signal is a measurement of αsyn self-association [32]–[34]. Fluorescence energy resonance transfer (FRET) has also been used to quantify αsyn aggregation by fusing two fluorophores to the N- and C-terminals of αsyn [35]. BiFC and FRET, however, suffer from inherent limitations. Fusion of αsyn to highly stable chromophores or to large protein fragments can perturb αsyn folding and alter its misfolding-propensity. In addition, these techniques are not optimal to measure protein self-association because they fail to detect homotypic interactions.

In this study, we developed an expression system that allows detecting and quantifying soluble αsyn in living cells. We adapted a previously reported split GFP molecule specifically engineered to study protein solubility [36]. This GFP variant is cleaved into two unequal size fragments, a 15-amino acid “sensor” fragment and a large “detector” fragment, that spontaneously complement upon chemical interaction, giving rise to a fluorescence signal [36]. αsyn was fused to the sensor fragment, which has minimal effect on the folding and solubility of its fusion partners and can therefore be used as a sensor of αsyn solubility. The resulting αsyn fusion protein was co-expressed with the large detector fragment in cell cultures. Fluorescent complementation is directly proportional to αsyn solubility as it occurs only if the sensor fragment escapes aggregation and is accessible to the detector fragment. The fluorescence of cells expressing wild type αsyn was compared to that of cells expressing αsyn variants with different aggregation properties: A53T αsyn, a C-terminal truncation variant (αsyn123), and a rationally designed triple proline mutant (A30P, A56P and A76P) with low propensity to aggregate (TP αsyn). Cell fluorescence was also evaluated upon inhibition of proteasomal degradation and was observed to correlate with αsyn solubility as predicted from *in vitro* studies. Our results indicate that this method provides a robust platform to quantify αsyn solubility in living cells and can be used to study αsyn sequence specificity and to monitor the influence of the cell folding network on αsyn aggregation.

## Results

### Quantification of αsyn Solubility using the αsyn-split GFP Assay

To study αsyn solubility in living cells we adapted a previously reported assay based on split GFP complementation [36]. In this assay, GFP is split into two moieties, GFP_1–10_, the bulk of the β-barrel (detector fragment), and GFP_11_, a 15-amino acid β-sheet (sensor fragment). GFP fragment complementation was shown to be inversely proportional to aggregation by comparing sequential expression and co-expression of GFP_11_-tagged proteins and GFP_1–10_ [36]. The small GFP_11_ tag was previously shown not to affect the folding of the fusion protein [36], [37] and was therefore fused to the C-terminal of αsyn in this study. The large GFP_1–10_ fragment was co-expressed with αsyn-GFP_11_ in the cytoplasm of mammalian cells. We hypothesized that if αsyn is maintained in a soluble state, the GFP_11_ tag is exposed to the solvent and can complement with GFP_1–10_, giving rise to a fluorescence signal. On the other hand, αsyn aggregation would preclude accessibility of GFP_11_ to GFP_1–10_, thus preventing fluorescence complementation. Hence, GFP fluorescence is expected to be proportional to αsyn solubility.

**Figure 1:**
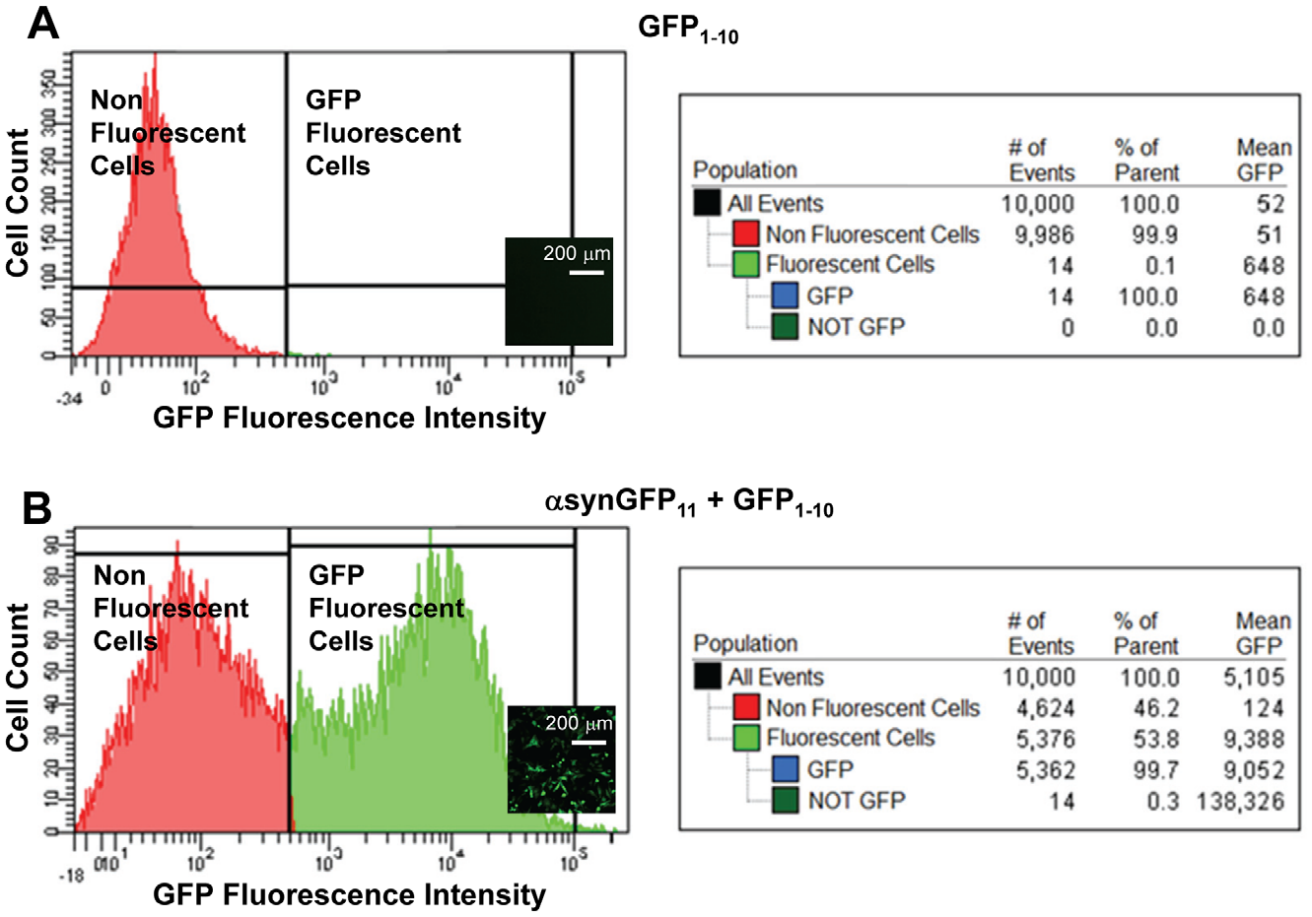
The αsyn-split GFP system enables quantification of soluble αsyn in HeLa cells. HeLa cells were transfected with αsyn-GFP_11_ and GFP_1–10_. Fluorescence was measured with a flow cytometer and live cells were imaged using fluorescence microscopy. (**A**) Representative fluorescence histogram (left panel) and data analysis (right panel) of cells expressing GFP_1–10_ only. Over 99% of the cell population does not display fluorescence. A representative image of live cells is reported in the figure inset. (**B**) Fluorescence histogram (left panel) and data analysis (right panel) of cells co-expressing αsyn-GFP_11_ and GFP_1–10_. Over 50% of the cell population display GFP fluorescence. A representative fluorescence microscopy image is reported in the figure inset. Scale bars represent 200 µm.

HeLa cells were transfected for the expression of αsyn-GFP_11_ and GFP_1–10_ and GFP fluorescence was evaluated by flow cytometry and fluorescence microscopy (Figure 1). As expected, cells expressing only GFP_1–10_ did not display detectable fluorescent signal (Figure 1A), whereas cells co-expressing αsyn-GFP_11_ and GFP_1–10_ exhibited GFP fluorescence when tested 18 hrs post transfection (Figure 1B). Fluorescence microscopy validated these results (Figure 1, inset), confirming that cell fluorescence is due to GFP fragment complementation. To ensure that the intensity of the fluorescence signal is not limited by the amount of GFP_1–10_ available for complementation with GFP_11_ and is therefore an accurate measurement of the concentration of soluble αsyn, a series of experiments were conducted in which increasing concentrations of plasmid encoding for GFP_1–10_ were used in the transfection procedure. A GFP_1–10_ to GFP_11_ ratio of 2∶1 was sufficient to ensure that fluorescent complementation is not limited by the concentration of GFP_1–10_ but rather depends on the amount of soluble αsyn-GFP_11_ (data not shown), in agreement with previously published work [36], [37]. This ratio of plasmid concentrations was used for all subsequent experiments.

**Figure 2:**
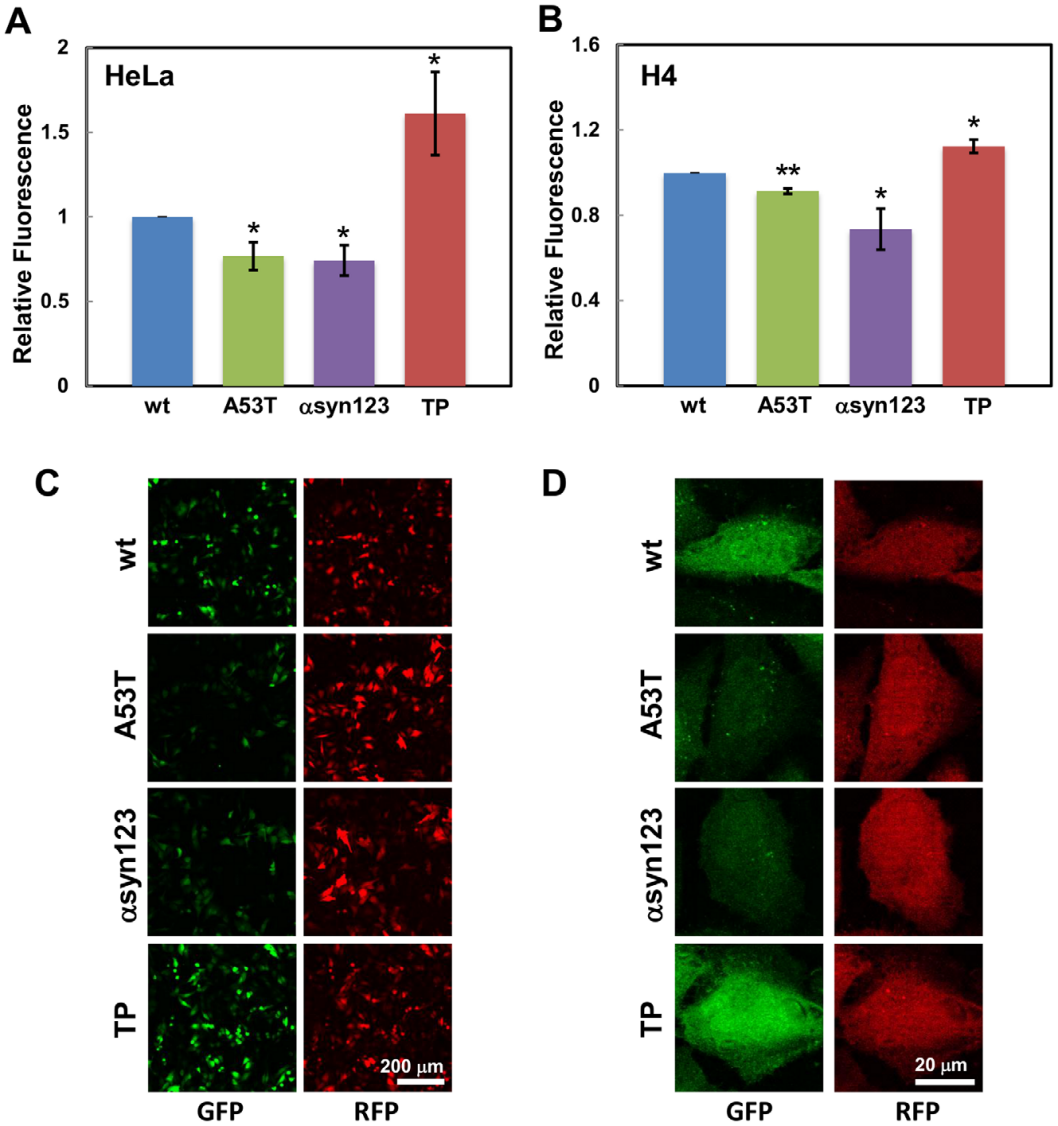
Mutations in αsyn gene sequence affect protein solubility. The fluorescence of cells expressing the αsyn-split GFP system was measured by flow cytometry and fluorescence microscopy. (**A**) Quantitative analysis of GFP fluorescence of HeLa cells expressing αsyn-GFP_11_+ GFP_1–10_ (blue), A53T αsyn-GFP_11_+ GFP_1–10_ (green), αsyn123-GFP_11_+ GFP_1–10_ (purple) and TP αsyn-GFP_11_+ GFP_1–10_ (red). (**B**) Quantitative analysis of GFP fluorescence of H4 cells expressing αsyn-GFP_11_ (blue), A53T αsyn-GFP_11_+ GFP_1–10_ (green), αsyn123-GFP_11_+ GFP_1–10_ (purple) and TP αsyn-GFP_11_+ GFP_1–10_ (red). Fluorescence measurements were normalized to fluorescence of cells expressing wild type αsyn-GFP_11_. *p<0.05; **p<0.005. Data points are reported as mean ± S.E.M. (n = 3). (**C**–**D**) Representative fluorescence microscopy images of HeLa cells expressing αsyn-GFP_11_+ GFP_1–10_ (blue, first row), A53T αsyn-GFP_11_+ GFP_1–10_ (green, second row), αsyn123-GFP_11_+ GFP_1–10_ (purple, third row) and TP αsyn-GFP_11_+ GFP_1–10_ (red, fourth row) at 20X (C) and 100X (D) magnification. GFP fluorescence is shown in the left column and mCherry fluorescence is shown in the right column. Scale bar represents 200 µm (C) and 20 µm (D).

Next, we compared wild type αsyn to three αsyn variants - A53T αsyn, TP αsyn and a C-terminal truncation mutant consisting of amino acids 1–123 (αsyn123). A53T αsyn was shown to aggregate faster than wild type αsyn in cells and *in vitro* [9], [10], [17]. The truncated αsyn123 has a shortened proline rich C terminal region, making it prone to aggregation in *in vitro* studies [18]–[20]. TP αsyn contains three proline substitutions (A30P, A56P and A76P) that disrupt the protein's ability to form aggregates *in vitro*, therefore preventing the formation of fibrils even after two weeks of incubation [24]. The solubility and aggregation propensity of TP αsyn in living cells, however, is not known. The mutations were introduced in the αsyn-GFP_11_ encoding gene. HeLa cells were transfected with three plasmid encoding the αsynGFP_11_ variants, GFP_1–10_, and mCherry, a highly photostable red fluorescent protein mutant [38] (a gracious gift from Dr. Jonathan Silberg, Rice University), used here as a transfection control. Cells were cultured for 18 hrs and GFP fluorescence measured by flow cytometry. Cells expressing TP αsyn exhibited 50% higher fluorescence than cells expressing wild type αsyn, whereas, GFP fluorescence was 25% lower in cells expressing A53T and αsyn123 (Figure 2A). Results obtained using HeLa cells suggest that the αsyn-split GFP assay can be used to quantify αsyn solubility in living cells. To validate the use of the αsyn-split GFP assay in a cell type more relevant to study the phenotype associated with PD cellular pathogenesis, these experiments were repeated using neuroglioma (H4) cells. As shown in Figure 2B, H4 cells expressing A53T αsyn and αsyn123 exhibited significantly lower fluorescence than wild type αsyn, while GFP fluorescence was significantly higher in H4 cells transfected with TP αsyn, confirming the results obtained in HeLa cells.

Fluorescence microscopy images of HeLa cells expressing the αsyn-split GFP system are reported in Figure 2C–D and include detection of GFP fluorescence (left column) and detection of mCherry fluorescence (right column). GFP fluorescence was observed to decrease in cells expressing A53T αsyn and αsyn123 and to increase in cells expressing TP αsyn, confirming results obtained with flow cytometry. The intensity of mCherry fluorescence, however, did not change in cells expressing different αsyn variants, demonstrating that the differences in GFP fluorescence complementation detected are not due to differences in transfection or expression efficiency, but are rather due to GFP fluorescence complementation. These differences in GFP fluorescence complementation were equally observed upon visualization of the whole cell population (Figure 2C) as well as in individual cells (Figure 2D), confirming the results obtained from flow cytometry. In summary, these results demonstrate that A53T αsyn and αsyn123 have a higher propensity to form aggregates and, therefore, lead to lower fluorescence complementation than wt and TP αsyn.

### Inhibition of Proteasomal Degradation Lowers αsyn Solubility and Prevents GFP Fluorescence Complementation

**Figure 3:**
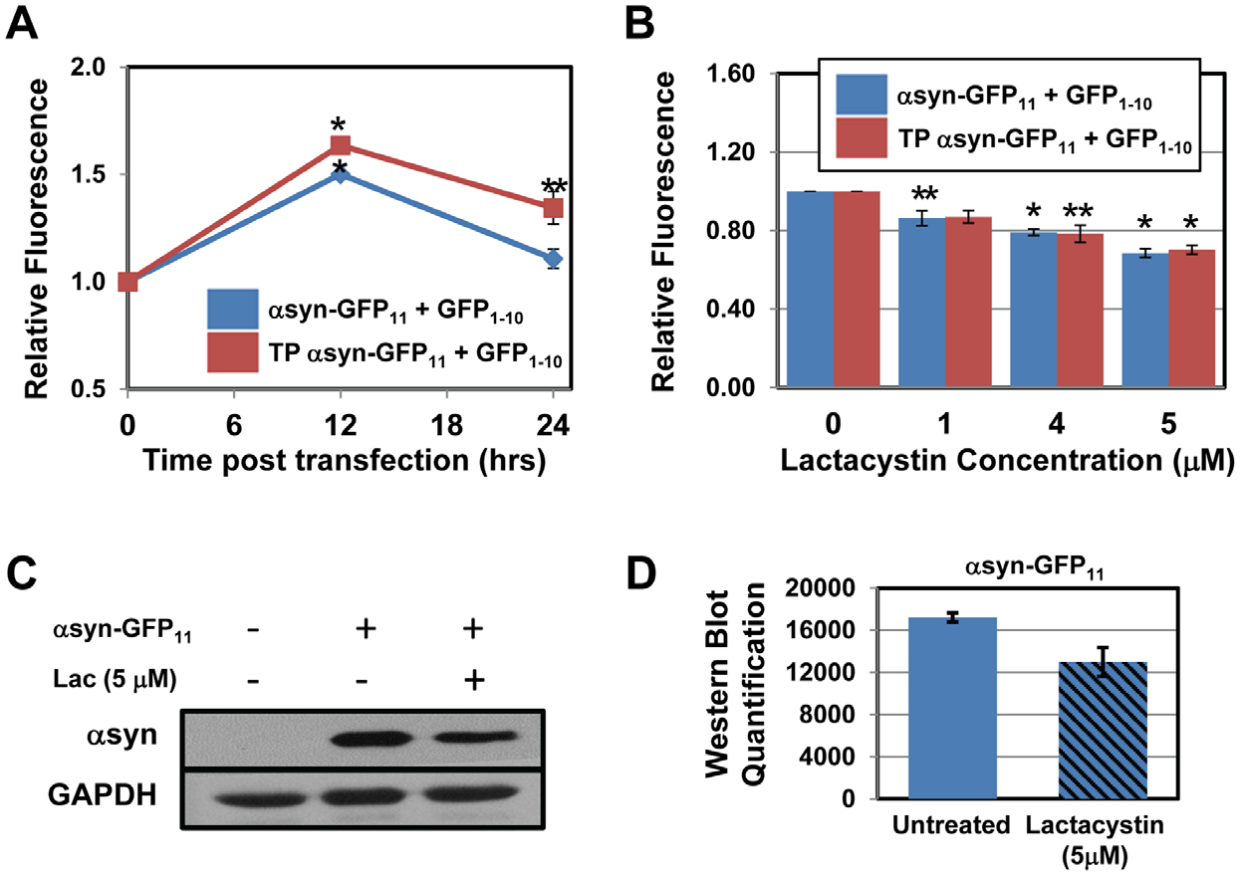
Inhibition of proteasomal degradation lowers αsyn solubility. (**A**) Quantitative analysis of GFP fluorescence of cells expressing αsyn-GFP_11_+ GFP_1–10_ (blue) and TP αsyn-GFP_11_+ GFP_1–10_ (red). Relative fluorescence was calculated by normalizing the fluorescence of cells 12 and 24 hrs post transfection to the fluorescence measured at 0 hr. *p<0.005; **p<0.05. (**B**) Relative fluorescence of cells expressing αsyn-GFP_11_ and GFP_1–10_ (blue) and TP αsyn-GFP_11_+ GFP_1–10_ (red), 24 hrs post transfection. Cells were incubated for 24 hrs with increasing concentrations of lactacystin (0–5 µM). Relative fluorescence was evaluated by normalizing the fluorescence of treated cells to the fluorescence of untreated cells. *p<0.01, **p<0.05. Data points are reported as mean ± S.E.M. (n = 3) (**C**) Representative western blot of cells expressing αsyn-GFP_11_, treated with lactacystin (5 µM) for 24 hrs, using αsyn-specific antibody. (**D**) Western blots band quantification of cells expressing αsyn-GFP_11_. Bands were quantified by NIH ImageJ analysis software. GAPDH was used as loading control.

Our results demonstrate that the αsyn-split GFP assay is a viable tool to study αsyn aggregation. This assay can be used to study the aggregation of naturally occurring αsyn variants and to predict the aggregation of rationally designed mutants such as TP αsyn. To further characterize the TP αsyn mutant, we tested its solubility in HeLa cells under cell culturing conditions that are expected to alter the solubility of misfolded, aggregation-prone proteins. To this end, we induced chemical inhibition of proteasomal degradation and investigated its effect on fluorescence complementation. Inhibition of the proteasome causes aberrant accumulation of misfolded proteins and formation of insoluble aggregates [39], [40]. Lactacystin is a highly selective proteasome inhibitor [41] that can easily penetrate the cell membrane and irreversibly block multiple hydrolytic activities in the proteasome [42]. HeLa cells expressing GFP_1–10_ and either αsyn-GFP_11_ or TP αsyn-GFP_11_ were treated with a range of concentrations of lactacystin for 24 hrs and GFP fluorescence was measured by flow cytometry. As shown in Figure 3A, cells expressing TP αsyn exhibited 10% and 21% higher fluorescence than cells expressing wild type αsyn after 12 and 24 hrs of incubation, respectively. Upon treatment with lactacystin, we observed a decrease in GFP fluorescence in a concentration dependent manner. Specifically, cells expressing αsyn wild type displayed 68% fluorescence of untreated cells upon treatment with 5 µM lactacystin and cells expressing TP αsyn displayed 70% fluorescence under the same conditions (Figure 3B). These results suggest that proteasomal inhibition, by causing an increase in αsyn aggregation, results in lowered GFP fluorescence complementation. Thus, this assay can be used to monitor the influence of the folding and degradation machinery on αsyn solubility. It should be noted that even though lactacystin treatment caused similar changes in fluorescence in cells expressing wild type αsyn and TP αsyn relative to untreated cells, the absolute fluorescence of cells expressing TP αsyn was significantly higher than that of cells expressing αsyn wild type (Figure S1), as reported before (Figure 2 & 3A).

In order to confirm that the loss of GFP fluorescence observed upon lactacystin treatment is due to increase in αsyn aggregation, αsyn solubility was investigated by Western blot. HeLa cells expressing αsyn-GFP_11_ were incubated with lactacystin (5 µM) for 24 hrs. The soluble protein fraction was collected and analyzed using an αsyn-specific antibody. Lactacystin-induced proteasome inhibition was observed to result in approximately 25% decrease in soluble αsyn (Figure 3C and 3D). This data indicates that the decrease in fluorescence complementation due to lactacystin treatment can be attributed to a decrease in soluble αsyn-GFP_11_.

### Fluorescence Complementation Inversely Correlates with the Formation of Cellular Aggregates

**Figure 4:**
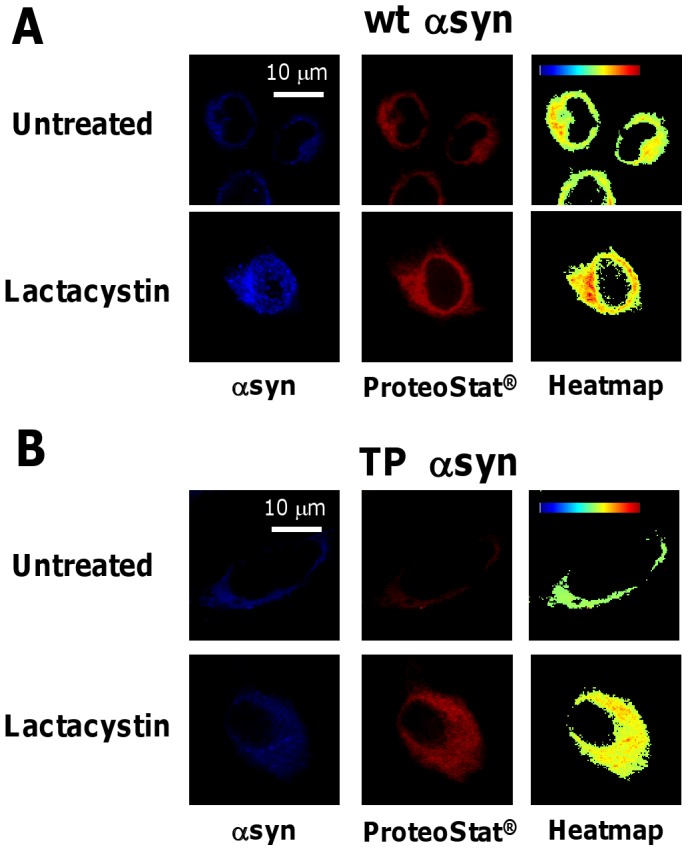
Inhibition of proteasomal degradation enhances αsyn aggregation. Immunofluorescence microscopy of cells expressing (**A**) αsyn-GFP_11_ and GFP_1–10_ and (**B**) TP αsyn-GFP_11_ and GFP_1–10_. Cells were treated with lactacystin (5 µM) for 24 hrs. Co-localization intensity of αsyn (blue, column 1) and ProteoStat® dye (red, column 2) is displayed in the form of co-localization heat maps (column 3). Hot colors represent positive co-localization and cold colors represent negative co-localization. Scale bars represent 10 µm.

To examine the correlation between fluorescence complementation and αsyn aggregation, we evaluated the formation of aggregates using immunofluorescence microscopy. Cells were cultured under conditions that gave rise to maximal change in GFP complementation and analyzed by immunofluorescence microscopy. Specifically, HeLa cells were transfected for the expression of GFP_1–10_ and either αsyn-GFP_11_ or TP αsyn-GFP_11_ and treated with lactacystin (5 µM) for 24 hrs. αsyn accumulation into cellular aggregates was detected using an antibody specific for αsyn (Figure 4, column 1, blue) and the ProteoStat® dye (Figure 4, column 2, red), a 488-nm excitable red fluorescent molecule that specifically interacts with denatured proteins within aggresomes [43]. Images showing co-localization of αsyn and the aggregate-specific dye were analyzed with NIH ImageJ software to obtain heatmaps (Figure 4, column 3). To quantify the aggregation of αsyn, co-localization events were counted and averaged over three independent experiments. The extent of co-localization was evaluated by analyzing the image heatmaps based on the color scale reported in Table 1 as described in the Materials and Methods. Our analysis revealed that the degree of αsyn aggregation induced by lactacystin treatment depends on αsyn sequence. Specifically, cells expressing αsyn-GFP_11_ display a 3-fold increase in αsyn aggregation upon treatment with lactacystin, while cells expressing TP αsyn-GFP_11_ exhibit only a 1.5-fold increase (Table 1, high aggregation). The extent of aggregation detected from fluorescence microscopy studies (Figure 4) inversely correlates with measurements of cell fluorescence obtained by flow cytometry (Figure 3). We therefore concluded that the decrease in fluorescence complementation observed in cells treated with lactacystin can be attributed to the increase in αsyn aggregation caused by inhibition of proteasomal degradation. Furthermore, the higher fluorescence complementation observed in cells expressing TP αsyn compared to wild type αsyn is a direct result of its lower rate of aggregation.

**Table 1:** ProteoStat® co-localization assay.

	αsyn						TP αsyn					
αsyn aggregation	Untreated			Lactacystin			Untreated			Lactacystin		
**Low** a	52.0	±	4.5	57.8	±	15.0	33.4	±	6.2	45.7	±	12.0
**High** b	9.8	±	4.6	28.9	±	9.2	7.1	±	4.0	10.8	±	3.0

a Low co-localization – 35 to 60, yellow pixels.

b High co-localization – 0 to 35, red pixels.

## Discussion

Aggregation of αsyn into proteinaceous inclusions [2] has been repeatedly associated with the development of PD pathogenesis [11], [44]. Therefore, there is an urgent need to understand the molecular mechanisms underlying αsyn misfolding and aggregation in living cells. Currently available methods to study aggregation in cell cultures, including the use of GFP fusions, BiFC and FRET, present a number of limitations mainly associated with the use of reporter molecules that alter αsyn misfolding and aggregation pathway [32], preclude rapid and high-throughput quantification and, most importantly, do not afford reliable distinction between soluble and insoluble pools of αsyn [45]. In this study, we report the use of a split GFP assay based on the detection of fluorescent complementation [36], previously reported for quantification of protein solubility *in vitro* [36], and in bacterial and mammalian cells [37]. The GFP variant used in this assay is split into a small “sensor” fragment, which was fused to αsyn in this study, and a large “detector” fragment. αsyn aggregation precludes accessibility of the sensor fragment to the detector fragment for fluorescence complementation. We demonstrated here that the αsyn-split GFP expression system provides a reliable tool to quantify αsyn solubility in living cells.

We investigated the utility of the αsyn-split GFP assay to study the relationship between αsyn sequence and its rate of aggregation in living cells. Mutations in the αsyn-encoding gene have been associated with the development of early onset familial cases of PD [6]–[8]. αsyn C-terminal truncations were observed to accumulate in LB [20], [46], [47]. The aggregation properties of naturally occurring and rationally designed αsyn mutants have been extensively characterized *in vitro* [24], [48], [49]. To evaluate the use of the αsyn-split GFP assay to study how αsyn sequence specificity affects protein aggregation, we tested a rationally designed variant (TP αsyn) known to resist aggregation *in vitro* [24]. We compared the fluorescence of cells expressing TP αsyn to that of cells expressing wild type αsyn, A53T αsyn and a truncated αsyn variant (αsyn123). We observed a significant increase in fluorescence in cells expressing TP αsyn compared to cells expressing wild type αsyn, demonstrating higher solubility of this αsyn variant in cell cultures. On the other hand, cells expressing the A53T mutant and the truncation mutant αsyn123 exhibited significantly lower fluorescence than cells expressing wild type αsyn, suggesting that these variants aggregate at higher rate and that aggregation lowers GFP fragment complementation and fluorescence. These results indicate that the αsyn-split GFP assay can be used to quantify the effect of mutations in αsyn-encoding gene on the protein aggregation propensity in living cells.

We also investigated whether the αsyn-split GFP assay can be used to study the impact of environmental factors that alter the efficiency of the folding quality control system on αsyn solubility. Although the causes of PD are far from understood, studies have shown that changes in the cellular environment such as oxidative stress and inflammation are involved in the progression of the disease [50]. Inducing oxidative stress or inflammation was shown to increase αsyn aggregation and αsyn-induced cytotoxicity [51], [52]. Furthermore, the accumulation of αsyn has also been associated with impairment of the proteasome [14]–[16]. In this study, proteasomal inhibition was chemically induced in cells expressing the αsyn-split GFP system and observed to lower fluorescence complementation, demonstrating that proteasome dysfunction lowers αsyn solubility.

Finally, we showed that the intensity of fluorescence of cells expressing the αsyn-split GFP system is inversely proportional to the extent of αsyn aggregation. Analysis of co-localization between αsyn and an aggregate-specific dye revealed that the increase in fluorescent signal measured correlates with the decrease in aggregate formation. These results demonstrate that the αsyn-split GFP assay can be used to investigate cell treatments that affect protein aggregation and that it will potentially enable molecular screenings for the discovery of compounds that modulate αsyn aggregation.

In summary, our results show that the αsyn-split GFP assay allows to quantitatively measure the solubility of αsyn in living cells. Furthermore, we demonstrated that this assay can be used to study the aggregation properties of αsyn mutants in cell cultures and elucidate the effects that modifiers of cellular protein folding have on αsyn aggregation.

## Methods

### Reagents, Cell Lines, and Media

Lactacystin was purchased from Cayman Chemicals. Cell culture media were purchased from Gibco and Invitrogen. Fetal bovine serum (FBS) was purchased from Atlanta Biologicals. JetPrime™ transfection kit was purchased from Polyplus Transfection. Proteostat® Aggresome Detection Kit was purchased from Enzo Life Sciences.

HeLa cells (ATCC) were grown in MEM (GIBCO) supplemented with 10% heat-inactivated FBS and 1% PSQ and maintained at 37°C and 5% CO_2_. Human H4 neuroglioma cells (HTB-148, ATCC) were cultured in high glucose DMEM (Invitrogen) supplemented with 10% heat-inactivated FBS, 1% PSQ, 4 mM L-Glutamine, and 1 mM sodium pyruvate, and maintained at 37°C and 5% CO_2_. Cell medium was replaced every 3 to 4 days and monolayers of cells were passaged upon reaching about 90% confluency.

### Plasmids and Transient Transfections

pCMV-mGFP Cterm S11 Neo Kan and pCMV-mGFP 1–10 Hyg Amp vectors were obtained from Theranostech, Inc. The sequence encoding for GFP_1–10_ was amplified from pCMV-mGFP_1–10_ Hyg Amp by PCR using the primers listed in Table S1 and subcloned into pcDNA4/TO (Invitrogen) using the KpnI and XhoI restriction sites, giving rise to pcDNA4/TO/GFP_1–10_. The cDNA encoding for α-syn was amplified from pcDNA6.2+αsyn-emGFP plasmid (lab collection) using the primers listed in Table S1 and cloned into the plasmid pCMV-mGFP Cterm S11 Neo Kan using XhoI and AgeI restriction sites, giving rise to pCMV-mGFP/αsyn-GFP_11_. The A53T substitution carrying mutant αsyn was constructed using the QuikChange® Site-Directed mutagenesis kit (Strategene) and KAPA HiFi HotStart PCR kit (Kapa Biosystems) following manufacturers’ protocols and using primers listed in Table S1, giving rise to pCMV-mGFP/A53Tαsyn-GFP_11_.The gene encoding for the αsyn mutant containing A30P, A56P, and A76P substitutions was constructed using the same procedure, using primers listed in Table S1, giving rise to pCMV-mGFP/TP αsyn-GFP_11_. The sequence of the truncated αsyn gene was amplified using pCMV-mGFP/αsyn-GFP_11_ as template and primers listed in table S1. The PCR product was cloned into the empty pCMV-mGFP Cterm S11 Neo Kan plasmid using XhoI and AgeI restriction sites, giving rise to pCMV-mGFP/αsyn123-GFP_11_.

Transfections were conducted in 6-well plates. 10^4^ cells were seeded in each well of a 6-well plate and plates were incubated for 24 hrs at 37°C. Transient transfections were performed using the JetPrime™ DNA transfection kit (Polyplus Transfection) according to the manufacturer’s procedures.

### GFP Complementation Analyses

HeLa or H4 cells were plated in 6-well plates and incubated for 24 hrs at 37°C. The media was removed and replaced with fresh media containing 0.33 µg of vectors encoding for wild type αsyn, A53T αsyn, TP αsyn or αsyn123 and 0.67 µg of pcDNA4/TO/GFP_1–10_ per well and transfected as described above. Transfection reactions were incubated for 16 hrs, at which point the media was replaced again. Cells were then collected and fluorescence was measured using a flow cytometer (FACSCanto™ II, BD Biosciences).

### Fluorescence Microscopy Analysis

HeLa cells were seeded on glass coverslips in 6-well plate and incubated for 24 hrs at 37°C. The media was removed and replaced with fresh media containing 0.33 µg/well of vectors encoding for wild type αsyn, A53T, TP αsyn or αsyn123, 0.67 µg/well of pcDNA4/TO/GFP_1–10_ and 0.2 µg/well of plasmid encoding for mCherry. The transfection reactions were incubated for 16 hrs, at which point they were washed with 0.1% Tween-20/PBS and fixed with 4% paraformaldehyde for 30 min. The coverslips were mounted on glass slides for fluorescence microscopy. The slides were imaged using an Olympus IX81 confocal microscope and analyzed using proprietary Fluoview software.

### Western Blot Analysis

HeLa cells were plated in 6-well plates and incubated for 24 hrs at 37°C. The media was removed and replaced with fresh MEM media containing 0.5 µg of pCMV-mGFP/αsyn-GFP_11_ per well and transfected as described above. Cells were lysed with complete lysis-M buffer (Roche) for 30min on ice with gentle rocking. The protein concentration was determined by Bradford assay (Pierce), and each sample was diluted to the same protein concentration. Proteins were separated by 12% SDS-polyacrylamide gels and transferred to a nitrocellulose membrane. Membranes were incubated with primary antibodies (mouse anti-α-syn (Sigma-Aldrich) and rabbit anti-GAPDH (Santa Cruz Biotechnology)) and appropriate secondary antibodies (HRP conjugated goat anti-rabbit and goat anti-mouse antibodies (Santa Cruz)). Blots were visualized using Millipore Luminata Forte HRP chemiluminescent substrate (Fisher) and quantified using NIH ImageJ software.

### Immunofluorescence and Co-localization Analyses

Cells were seeded on glass coverslips in 6-well plate, transfected, incubated in the presence of small molecules for 24 hrs and fixed with 4% paraformaldehyde for 30 min. Cells were permeabilized with a 0.5% Triton X-100, 0.6% 0.5 M EDTA solution in Assay buffer (Proteostat® Aggresome Detection Kit, Enzo) for 30 min on ice, followed by incubation in 8% BSA (blocking buffer) for 1 hr at room temperature. Cells were then incubated for 1 hr with primary antibody (mouse anti-αsyn, Sigma-Aldrich), washed with 0.1% Tween-20/PBS, and incubated with secondary antibody (Dylight 649 Goat anti-mouse from KPL). Cells were washed again and incubated with ProteoStat® dye (Proteostat® Aggresome Detection Kit, Enzo) for 30 min in the dark. The coverslips were mounted on glass slides for fluorescent microscopy. The slides were imaged using an Olympus IX81 confocal microscope and analyzed using proprietary Fluoview software.

Co-localization of αsyn with the ProteoStat® dye was evaluated using the ImageJ plugin Co-localization Colormap [53]. Results are reported in the form of co-localization heatmaps where hot colors represent positive co-localization, and cold colors represent negative co-localization. The co-localization heatmaps were analyzed using the ImageJ plugin Threshold Colour, which allows RGB images to be filtered based on the hue, saturation, and brightness of the pixels. Images were filtered to display RGB color hues as follows: high co-localization (RGB hue: 0–35, red pixels) and low co-localization (RGB hue: 36–60, yellow pixels). Pixels falling in the RGB hue range 60–255 were considered negative correlation and not evaluated in this study. For each sample, 85–120 cells were analyzed to count co-localization events.

### Statistical Analysis

All data are presented as mean ± S.E.M. Statistical significance was calculated using a two-tailed Student’s t test. Values were considered significantly different when *p* was <0.05.

## Supporting Information

Figure S1 **Effect of** **inhibition of proteasomal degradation on αsyn solubility and split GFP fluorescence complementation.** Representative plot of absolute GFP fluorescence in cells expressing αsyn-GFP_11_ and GFP_1–10_ (blue) and TP αsyn-GFP_11_ and GFP_1–10_ (red). Cells were incubated for 24 hrs with increasing concentrations of lactacystin (0–5 µM).
(TIF)

Table S1 **Primers used to construct GFP_1–10_, wt αsyn-GFP_11_, A53T αsyn-GFP_11_, TP asyn-GFP_11_, and αsyn123-GFP_11_.**
(DOC)
