# Supplementary figures and images for: Quantitative Analysis of α-Synuclein Solubility in Living Cells Using Split GFP Complementation
Source: PLoS One. 2012 Aug 22;7(8):e43505. doi: 10.1371/journal.pone.0043505 (PMC3425482; doi:10.1371/journal.pone.0043505)

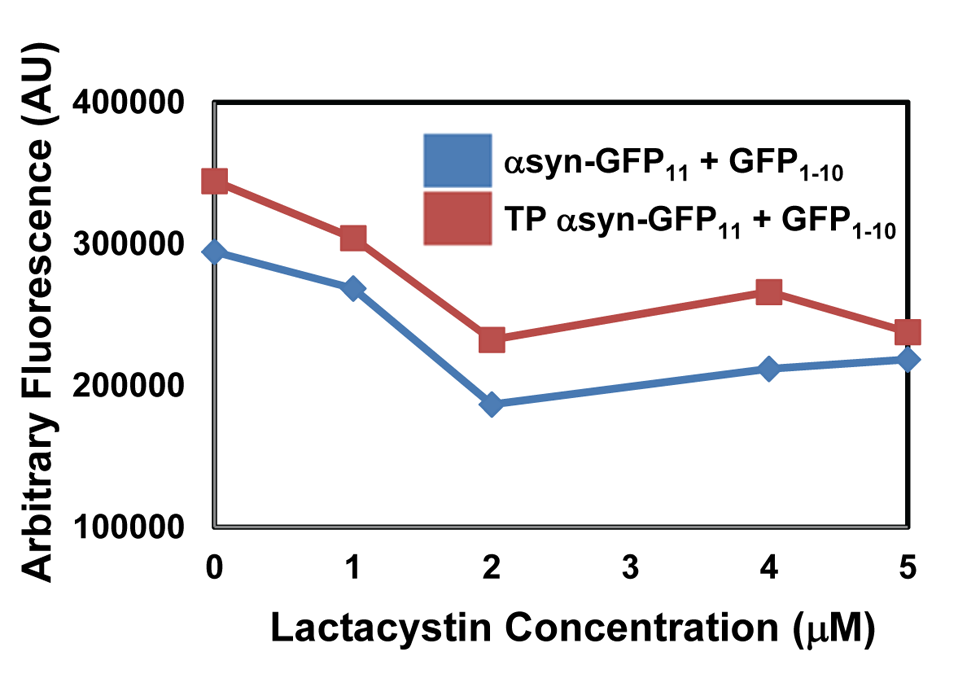

Supplement: Figure S1 — Effect of inhibition of proteasomal degradation on αsyn solubility and split GFP fluorescence complementation. Representative plot of absolute GFP fluorescence in cells expressing αsyn-GFP11 and GFP1–10 (blue) and TP αsyn-GFP11 and GFP1–10 (red). Cells were incubated for 24 hrs with increasing concentrations of lactacystin (0–5 µM). (TIF) [file pone.0043505.s001.tif]
