# Supplementary material for: Quantitative Analysis of α-Synuclein Solubility in Living Cells Using Split GFP Complementation
Source: PLoS One. 2012 Aug 22;7(8):e43505. doi: 10.1371/journal.pone.0043505 (PMC3425482; doi:10.1371/journal.pone.0043505)
Supplement: Table S1 — Primers used to construct GFP1–10, wt αsyn-GFP11, A53T αsyn-GFP11, TP asyn-GFP11, and αsyn123-GFP11. (DOC) [file pone.0043505.s002.doc]

**Table S1. Primers used to construct GFP_1-10_, wt αsyn-GFP_11_, A53T αsyn-GFP_11_, TP asyn-GFP_11_, and αsyn123-GFP_11_.**

| **Gene** | **Primer** | **Sequence** |
| --- | --- | --- |
| **αsyn-GFP_11_** | asyn_F1 | 5’-GCGCGCCTCGAGATGGATGTATTCATGAAAGGACTTTCAAAGGCC-3' |
|  | GFP11_R1 | 5'-CTATATACCGGTTTGGCTTCAGGTTCGTAGTCTTGATACCC-3' |
| **GFP_1-10_** | GFP1-10_F1 | 5'-GCTATGGTACCATGGTTTCGAAAGGCGAGGAGCTGTTC-3' |
|  | GFP1-10_R1 | 5'-CGCGCCTCGAGTTATTTCTCGTTTGGGTCTTTGCTCAG-3' |
| **A53T αsyn-GFP_11_** | A53T_F1 | 5’-GTGGTGCATGGTGTGACAACAGTGGCTGAGAAGACCAAAGAG-3' |
|  | A53T_R1 | 5'-CTCTTTGGTCTTCTCAGCCACTGTTGTCACACCATGCACCAC-3' |
| **αsyn123-GFP_11_** | GFP11_F1 | 5’-GCGCGCCTCGAGATGGATGTATTCATGAAAGGACTTTCAAAGGCC-3' |
|  | Syn123_R1 | 5'-CTATATACCGGTTTCTCATTGTCAGGATCCACAGGCATATCTTCCAGAATTC-3' |
| **TP αsyn-GFP_11_** | A30P_F | 5’-CAGGGTGTGGCAGAAGCACCAGGAAAGACAAAAGAGGGTGTTCTC-3’ |
|  | A30P_R | 5’-GAGAACACCCTCTTTTGTCTTTCCTGGTGCTTCTGCCACACCCTG-3’ |
|  | A56P_F | 5’-CATGGTGTGGCAACAGTGCCTGAGAAGACCAAAGAG-3’ |
|  | A56P_R | 5’-CTCTTTGGTCTTCTCAGGCACTGTTGCCACACCATG-3’ |
|  | A76P_F | 5’-GTGGTGACGGGTGTGACACCAGTAGCCCAGAAGAC-3’ |
|  | A76P_R | 5’-GTCTTCTGGGCTACTGGTGTCACACCCGTCACCAC-3’ |
